# Inflammation, Apoptosis, and Fibrosis in Diabetic Nephropathy: Molecular Crosstalk in Proximal Tubular Epithelial Cells and Therapeutic Implications

**DOI:** 10.3390/cimb47110885

**Published:** 2025-10-24

**Authors:** Xuanke Liu, Chunjiang Zhang, Yanjie Fu, Linlin Xie, Yijing Kong, Xiaoping Yang

**Affiliations:** 1 Medical College, Shihezi University, Shihezi 832000, China; 13318230127@163.com (X.L.); 18099043257@163.com (C.Z.); 15916160307@163.com (Y.F.); 20252114027@stu.shzu.edu.cn (L.X.); 18449391960@163.com (Y.K.); 2 Key Laboratory of Prevention and Treatment of Central Asia High Incidence Diseases (Co-Construction), National Health Commission (NHC), Shihezi 832000, China

**Keywords:** diabetic nephropathy, proximal renal tubular epithelial cells, inflammation, apoptosis of cells, renal tubulointerstitial fibrosis, transforming growth factor-β1, treatment strategies

## Abstract

Diabetic nephropathy (DN) remains the leading cause of end-stage renal disease worldwide, with proximal tubular epithelial cells (PTECs) playing a central role in its pathogenesis. Under hyperglycemic conditions, PTECs drive a pathological triad of inflammation, apoptosis, and fibrosis. Recent advances reveal that these processes interact synergistically to form a self-perpetuating vicious cycle, rather than operating in isolation. This review systematically elucidates the molecular mechanisms underlying this crosstalk in PTECs. Hyperglycemia induces reactive oxygen species (ROS) overproduction, advanced glycation end products (AGEs) accumulation, and endoplasmic reticulum stress (ERS), which collectively activate key inflammatory pathways (NF-κB, NLRP3, cGAS-STING). The resulting inflammatory milieu triggers apoptosis via death receptor and mitochondrial pathways, while apoptotic cells release damage-associated molecular patterns (DAMPs) that further amplify inflammation. Concurrently, fibrogenic signaling (TGF-β1/Smad, Hippo-YAP/TAZ) promotes epithelial–mesenchymal transition (EMT) and extracellular matrix (ECM) deposition. Crucially, the resulting fibrotic microenvironment reciprocally exacerbates inflammation and apoptosis through mechanical stress and hypoxia. Quantitative data from preclinical and clinical studies are integrated to underscore the magnitude of these effects. Current therapeutic strategies are evolving toward multi-target interventions against this pathological network. We contrast the paradigm of monotargeted agents (e.g., Finerenone, SGLT2 inhibitors), which offer high specificity, with that of multi-targeted natural product-based formulations (e.g., Huangkui capsule, Astragaloside IV), which provide synergistic multi-pathway modulation. Emerging approaches (metabolic reprogramming, epigenetic regulation, mechanobiological signaling) hold promise for reversing fibrosis. Future directions include leveraging single-cell technologies to decipher PTEC heterogeneity and developing kidney-targeted drug delivery systems. We conclude that disrupting the inflammation–apoptosis–fibrosis vicious cycle in PTECs is central to developing next-generation therapies for DN.

## 1. Introduction

Diabetic nephropathy (DN) is one of the most serious microvascular complications of diabetes and the leading cause of end-stage renal disease (ESRD) in the world. Its pathological features are glomerular sclerosis, renal tubulointerstitial fibrosis, and progressive loss of renal function [1]. Although traditional studies mostly focus on glomerular lesions, a large number of clinical and basic research studies in recent years have shown that tubulointerstitial injury plays a non-negligible role in the occurrence and development of DN and may even be more correlated with renal function decline than glomerular lesions [2]. This shift in understanding has led researchers to look at proximal tubule epithelial cells (PTECs), which make up more than 80% of the kidney’s volume and are not only the main operators of glucose reabsorption but are key integrators and amplifiers of DN pathological signals [3]. It is important to note that while glomerular components, particularly mesangial cells, are undeniably crucial in the early stages of DN—contributing to mesangial expansion and glomerulosclerosis—the proximal tubule has emerged as a pivotal site driving disease progression, especially in the context of tubulointerstitial fibrosis, which strongly correlates with declining renal function. PTECs are metabolically highly active and are primary targets of glucotoxicity, lipotoxicity, and protein overload. Their dysfunction initiates and propagates key pathological processes—inflammation, apoptosis, and fibrosis—that extend beyond the tubule to influence the glomerular compartment and the entire renal interstitium. For instance, injured PTECs release pro-inflammatory and pro-fibrotic factors that can exacerbate glomerular damage, and vice versa, creating damaging crosstalk.

In a hyperglycemic environment, PTECs excessively reabsorb glucose through Sodium-Glucose Cotransporter 2 (SGLT2), leading to intracellular metabolic disorders, triggering mitochondria (MT) dysfunction and oxidative stress outbreak [4,5]. This process activates a complex pathological network, including inflammatory responses, apoptosis, and fibrotic processes. It is worth noting that these three pathological processes do not exist in isolation but form a vicious circle through dynamic interaction: The inflammatory microenvironment is regulated by death receptor (DR) pathways such as Tumor Necrosis factor-alpha/Tumor Necrosis Factor receptor (TNF-α/TNFR) [6] and MT pathways (such as Bcl-2 family protein imbalance) which trigger apoptosis [7], and apoptotic cells release damage-associated molecular patterns (DAMPs) such as High Mobility Group Box 1 (HMGB1) activate Nuclear Factor-kappa B (Nf-κB) through Toll-like receptors (TLRs) and the NF-κB pathway, further amplifying the inflammatory response [8,9]. Transforming Growth factor-beta (TGF-β1), a key factor in transforming growth factor-beta, plays an important role in inflammation and apoptosis. TGF-β1/Smad3 and Hippo signaling pathway-Yes-associated protein/Transcriptional coactivator with PDZ-binding motif (Hippo-YAP/TAZ) and other pathways drive epithelial–mesenchymal transition (EMT) and extracellular matrix (ECM) deposition, eventually leading to irreversible tubulointerstitial fibrosis [10,11]. The fibrotic microenvironment activates inflammatory and apoptotic signals through mechanical stress feedback, forming a self-reinforcing cycle [12,13]. The latest single-cell sequencing studies have revealed that PTECs show significant spatial and temporal heterogeneity in DN, and specific subsets (such as CD44hi/ITGB1hi cells) may act as a “stubborn stronghold” for promoting fibrosis and may promote the development of the disease to the terminal stage [14].

In recent years, with the in-depth understanding of the central role of PTECs in DN, therapeutic strategy has also shifted from traditional glycemic control to multi-target intervention targeting the inflammation–apoptosis–fibrosis network. In terms of Western drug research and development, new renoprotective drugs such as non-steroidal mineralocorticoid receptor antagonists (e.g., Finerenone) and SGLT2 inhibitors (e.g., Empagliflozin) have demonstrated renoprotective effects through clinical trials. The mechanism involves inhibition of NF-κB and NOD-like receptor family pyrin domain containing 3 (NLRP3) inflammasome [15,16]. At the same time, the synergism of multiple components of traditional Chinese medicine shows unique therapeutic potential. For example, flavonoid components (Quercetin and Myricetin) in Huangkui capsules can regulate Phosphoinositide 3-kinase/Protein kinase B (PI3K/Akt), Janus kinase 2/Signal transducer, and signal transduction simultaneously of the activator of transcription 3 (JAK2/STAT3), and TGF-β1/Smad3 intervened in the pathological processes of PTECs, such as inflammation, apoptosis, and fibrosis [17,18,19]. The Yishen Huashichi granule can reduce EMT and fibrosis of renal tubular epithelial cells by inhibiting the TGF-β1/Smad3 pathway [20]. Its active ingredients, such as Astragaloside IV and Ginsenoside Rg1, have clear anti-inflammatory and anti-oxidation effects [21,22]. These compound traditional Chinese medicines provide new strategies for the treatment of diabetic nephropathy with the advantages of multi-target and multi-pathway synergistic regulation. In addition, emerging strategies targeting energy metabolic reprogramming (such as glycolysis and oxidative phosphorylation imbalance) and epigenetic regulation (such as m6A modification) of PTECs also provide breakthrough directions for DN treatment [23].

However, there are still many unanswered questions about the pathological mechanism of DN. For example, the differences in molecular responses of PTECs between type 1 and type 2 diabetes mellitus, the dynamic evolution of the inflammation–apoptosis–fibrosis cascade, and how the mechanical mechanics of the microenvironment regulate cell phenotype transformation through the integrin-Yes-associated protein (YAP) signaling axis still need to be explored [24,25]. In addition, the long-term efficacy and safety of the existing treatment strategies still need to be supported by more clinical evidence. This article systematically reviews the interactive regulatory mechanism and treatment progress of PTECs in inflammation, apoptosis, and fibrosis in DN, aiming to provide a theoretical basis for the development of more precise targeted intervention strategies and ultimately improve the clinical outcome of DN patients.

## 2. Molecular Mechanism and Network Regulation of Inflammatory Response of PTECs

The inflammatory response of PTECs in DN is one of the core links in the occurrence and development of the disease, and its mechanism involves a complex regulatory network of multi-levels and multi-pathways. A high-glucose environment induces intracellular metabolic disorders, leading to reactive oxygen species (ROS) burst [26], accumulation of advanced glycation end products (AGEs) [27], and endoplasmic reticulum stress (ERS) [28] and other pathological changes, and also activates key inflammatory signaling pathways including NF-κB, NLRP3 inflammasome, Cyclic GMP-AMP Synthase-Stimulator of Interferon Genes (cGAS-STING) [29], and so on. These pathways not only act independently but also form a positive feedback loop through “crosstalk” to continuously amplify the inflammatory response and promote tubulointerstitial injury. In recent years, with the development of single-cell sequencing, proteomics, and gene editing technology, the spatiotemporal heterogeneity and microenvironment dependence of PTECs in inflammatory response have been gradually revealed. For example, specific PTEC subsets, such as CD44 or ITGB1 cells, show a stronger activation state in the initiation and maintenance of inflammation and may act as “inflammation amplifiers” to drive disease progression [30,31]. In addition, inflammatory mediators regulate the infiltration and activation of immune cells through autocrine and paracrine ways, further changing the local microenvironment and forming a vicious cycle that is difficult to break. Therefore, an in-depth understanding of the molecular mechanism and network regulation of the inflammatory response of PTECs will not only help to reveal the pathogenesis of DN but also provide a theoretical basis for the development of targeted therapeutic strategies.

### 2.1. Activation of ROS-Dependent Inflammatory Pathways

ROS are highly reactive molecules derived from oxygen, primarily generated from mitochondrial respiration and NADPH oxidase (NOX) family enzymes. Under hyperglycemia, excessive ROS production overwhelms cellular antioxidant defenses, leading to a state of oxidative stress.

**Figure 1:**
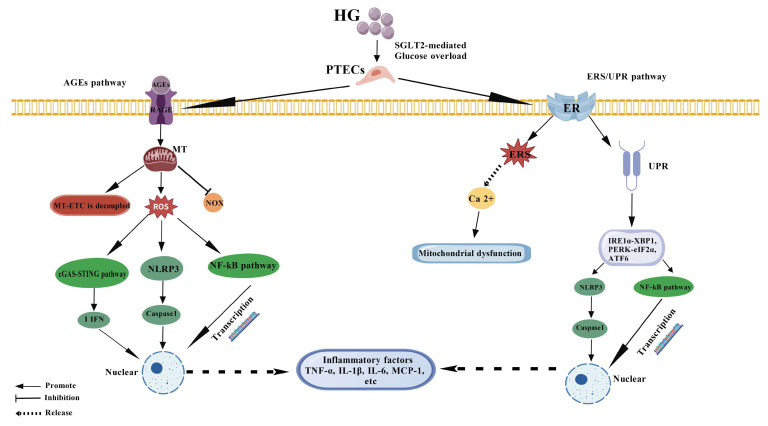
Molecular mechanisms underlying the inflammatory response in PTECs under hyperglycemic conditions. Hyperglycemia induces intracellular metabolic disturbances, leading to three key initiating events: (1) ROS burst originating from mitochondrial dysfunction and NOX activation; (2) accumulation of AGEs and their engagement with RAGE; (3) endoplasmic reticulum stress (ERS) and unfolded protein response (UPR). These events converge to activate pivotal pro-inflammatory signaling pathways, including NF-κB, the NLRP3 inflammasome, and cGAS-STING. The subsequent release of cytokines (e.g., TNF-α, IL-1β, IL-6, MCP-1) and DAMPs fosters a sustained inflammatory microenvironment, which further exacerbates renal injury in diabetic nephropathy. Solid arrows indicate activation or promotion; dashed arrows indicate translocation or release. ROS, reactive oxygen species; AGEs, advanced glycation end products; RAGE, receptor for AGEs; ER, endoplasmic reticulum; UPR, unfolded protein response; NOX, NADPH oxidase; MT, mitochondria; NF-κB, nuclear factor kappa-light-chain-enhancer of activated B cells; NLRP3, NOD-like receptor family pyrin domain containing 3; cGAS, cyclic GMP-AMP synthase; STING, stimulator of interferon genes.

The overproduction of ROS induced by high glucose is an important initiating factor for the inflammatory response of PTECs. ROS is mainly derived from the uncoupling of the MT electron transport chain, activation of NOX family, and accumulation of AGEs [32]. Recent studies have shown that MT ROS not only directly causes oxidative damage but also acts as second messengers to activate multiple pro-inflammatory signaling pathways. For example, ROS oxidative modification of protein kinase Cδ (PKCδ) further activate IκB kinase (IKK), leading to the degradation of IκBα and the release of NF-κB into the nucleus, initiating the transcription of TNF-α, Interleukin-6 (IL-6), Monocyte Chemoattractant Protein-1 (MCP-1), and other inflammatory factors [33,34]. In addition, ROS can directly activate the NLRP3 inflammasome, promote Cysteine-aspartic ase 1 (caspase-1)-mediated maturation and secretion of IL-1β and IL-6, and enhance inflammatory response [35]. For example, Bai et al. found that MT DNA (mtDNA) was released into the cytoplasm of PTECs due to oxidative damage under a high-glucose environment, which induced the production of type I interferon (IFN-β) by activating the cGAS-STING pathway and further amplifying the renal tubular inflammatory microenvironment [36]. This mechanism was validated in both diabetic mouse models and human renal tubular cell lines, suggesting that the mtDNA-cGAS-STING axis may be a key bridge connecting metabolic disorders and innate immune responses. The study by Jha et al. further revealed that NOX5 is highly expressed in diabetic kidneys, which activate the NLRP3 inflammasome and promote IL-1β release in a ROS-dependent manner, and deletion of NOX5 can significantly reduce renal tubular inflammation and fibrosis [37]. As summarized in (Figure 1), ROS serve as a central node, initiating these key pro-inflammatory cascades.

In addition, recent studies have found that ROS can also affect the expression of inflammatory genes by regulating epigenetic modifications, such as histone acetylation and DNA methylation [38,39]. For example, the elevation of ROS induced by high glucose can inhibit histone deacetylase (HDAC) activity, leading to increased histone acetylation levels in the promoter regions of pro-inflammatory genes and enhancing their transcriptional activity [40]. Taken together, ROS are not only effector molecules of oxidative stress but also a core node in the regulation of the inflammatory network, and specific inhibitors targeting ROS sources or downstream signals may have therapeutic potential.

### 2.2. Amplifier Effect of AGEs-RAGE Axis

AGEs are a heterogeneous group of compounds formed by the non-enzymatic glycation and oxidation of proteins, lipids, or nucleic acids. They accumulate under persistent hyperglycemia. The receptor for RAGE (Receptor for Advanced Glycation End-products, RAGE) is a multi-ligand pattern recognition receptor that transduces the pathogenic signals of AGEs.

AGEs accumulate in high-glucose environments and play an “amplifier” role in the inflammatory response of PTECs through binding to its receptor RAGE [41]. AGEs-RAGE interaction not only enhances ROS production but also directly activates multiple pro-inflammatory signaling pathways to form a positive feedback loop. Studies have shown that AGEs activate NOX4 after binding to RAGE [42], further increase ROS production, promote nuclear translocation of NF-κB through Toll-like receptor 4 (TLR4)/myeloid differentiation factor 88 (MyD88) axis, and upregulate the expression of inflammatory factors such as TNF-α and IL-6 [43,44]. In addition, AGEs-RAGE signaling enhanced the assembly and activation of the NLRP3 inflammasome and promoted caspase-1-dependent maturation of IL-1β [45]. In recent years, a number of studies have revealed the regulatory mechanism of the AGEs-RAGE axis in DN. For example, Zhu et al. found that RAGE depletion significantly reduced renal tubular inflammation and fibrosis in diabetic mice, and the mechanism was associated with inhibition of NF-κB and NLRP3 activation [46]. More importantly, it was also found that RAGE affected ROS generation by regulating MT dynamics (fusion/fission balance), suggesting a role of RAGE in regulating inflammatory response at the organelle level [47]. On the other hand, soluble RAGE (sRAGE), as an endogenous RAGE antagonist, competitively inhibits AGEs-RAGE signaling [48]. A clinical study by Nakamura et al. found that serum sRAGE levels in DN patients were negatively correlated with markers of renal tubular damage, such as KIM-1 and NGAL, suggesting that sRAGE may have a protective effect [49].

However, the dynamics of sRAGE in different stages of the disease and its regulatory mechanisms are not fully understood. Recent studies have also shown that AGEs can alter the physical and chemical properties of the ECM by modifying proteins such as collagen and laminin, which in turn activate the YAP/TAZ pathway through integrin signaling and promote inflammation and fibrosis [50,51]. Therefore, the AGEs-RAGE axis not only directly activates inflammatory signals but also indirectly regulates the inflammatory response of PTECs by changing the mechanical properties of the microenvironment. Multi-target interventions targeting this axis, such as RAGE antagonists, sRAGE analogs, or AGEs scavengers [52], have become a research hotspot in the treatment of DN. The pivotal role of the AGEs-RAGE axis in initiating and amplifying inflammation is depicted in (Figure 1).

### 2.3. Pro-Inflammatory Effects of ER Stress and Unfolded Protein Response

The endoplasmic reticulum (ER) is a crucial organelle for protein folding, maturation, and calcium storage. ERS occurs when the load of unfolded or misfolded proteins exceeds the ER’s processing capacity. To cope with ERS, cells activate the unfolded protein response (UPR), an adaptive signaling network.

ERS and UPR triggered by ERS play a key role in the inflammatory response of PTECs induced by high glucose [53]. High glucose causes accumulation of unfolded or misfolded proteins in the ER of PTECs, which activates three major branching pathways of the UPR: Inositol-requiring enzyme 1 αlpha-X-box binding protein 1 (IRE1α-XBP1) [54], PKR-like ER kinase-Eukaryotic Initiation Factor 2 αlpha subunit (PERK-eIF2α) [55], and Activating Transcription Factor 6 (ATF6) [56]. These pathways not only attempt to restore protein homeostasis but also switch to pro-inflammatory and pro-apoptotic signaling in response to sustained stress. In recent years, it has been found that there is wide crosstalk between UPR pathway and inflammatory signaling. For example, the IRE1α-XBP1 pathway can upregulate TNF-α, IL-1β and other inflammatory factors expression, promote Th17 cell infiltration, and exacerbate renal tubulointerstitial inflammation [57]. The PERK-eIF2α pathway activates ATF4 and C/EBP homologous protein (CHOP) to promote the release of IL-6 and TNF-α [58]. In addition, ERS can also promote PTECs apoptosis by activating apoptosis signal regulated kinase 1 (ASK1) -c-Jun N-terminal kinase (JNK) pathway and further aggravate kidney injury [59]. For example, Wang et al. showed that PERK/eIF2α/CHOP signaling promotes M1-type macrophage polarization and enhances renal tubular inflammation in diabetic nephropathy. Inhibition of PERK activity significantly reduced inflammatory factor levels and macrophage infiltration [60]. The study by Liu et al. found that the IRE1α/XBP1s signaling pathway mediates the inflammatory response, injury, and fibrosis of renal tubular epithelial cells by positively regulating the activation of the NLRP3 inflammasome in diabetic nephropathy [61]. As illustrated in (Figure 1), these ERS-UPR pathways converge to fuel the inflammatory cascade.

In addition, ERS also closely interacts with MT function. The endoplasmic reticulum and mitochondria are physically and functionally connected through MT-associated endoplasmic membranes (MAMs). ERS can lead to calcium ion release and MT calcium overload, further induce MT ROS production and membrane potential collapse, and amplify inflammatory and apoptotic signals [62,63]. In studies by Chen et al., the PERK/eIF2α/ATF4 signaling pathway mediates ER stress-induced acute lung injury, and 4-phenylbutyric acid (4-PBA)4-PBA can alleviate tissue injury by inhibiting this pathway. This mechanism suggests that targeting the PERK/eIF2α/ATF4 pathway or using 4-PBA may be a therapeutic strategy to alleviate ER stress and cell damage in hyperglycemia-induced PTEC injury [63]. Recent studies have also shown that ERS can affect inflammatory responses by regulating autophagic flux [64]. For example, persistent ERS inhibits autophagy initiation, leading to damaged organelles and protein accumulation, which further activates the NLRP3 inflammasome [65]. Therefore, targeting ERS-UPR signaling by using IRE1α or PERK inhibitors or modulating MAMs stability may become new strategies to alleviate inflammation in PTECs. The complex interplay between these initiating events and inflammatory signaling pathways is summarized (Figure 1).

## 3. The Triggering Pathway and Downstream Effects of PTECs Apoptosis

In the pathological process of DN, apoptosis of PTECs is not only a direct manifestation of the toxic effect of high glucose but also a core mechanism that promotes the transformation of the disease from early metabolic disorder to late fibrosis. Recent studies have gradually revealed that apoptosis of PTECs is far from a passive cell clearance process but actively participates in the dynamic events of microenvironment remodeling, inflammation amplification, and fibrosis progression [66,67]. With the development of single-cell transcriptomics, proteomics, and in vivo imaging techniques, researchers have gained a deeper understanding of the molecular pathways of apoptosis in PTECs and their role in multicellular dialog. A high-glucose environment induces caspase-dependent and caspase-independent cell death by activating exogenous (DR-mediated) and endogenous (MT and ERS mediated) apoptotic pathways, then releases DAMPs and extracellular vesicles (EVs), activates immune response, and promotes the activation of fibroblasts, forming a vicious cycle of “apoptosis-inflammation-fibrosis” [68,69]. Notably, apoptosis of PTECs in DN shows marked spatial–temporal heterogeneity and microenvironment dependence, and differences in the response of different cell subsets to apoptotic stimuli may determine the regionality and irreversibility of disease progression. Therefore, in-depth analysis of the mechanism of PTECs apoptosis not only helps to understand the pathological nature of DN but also provides new ideas for the development of targeted intervention strategies.

### 3.1. Exogenous Apoptotic Pathway: Signaling Cascade Mediated by Death Receptor

The extrinsic apoptotic pathway is initiated outside the cell through the engagement of transmembrane DR by their specific ligands. This pathway is particularly potent in inflammatory environments where these ligands are abundant.

**Figure 2:**
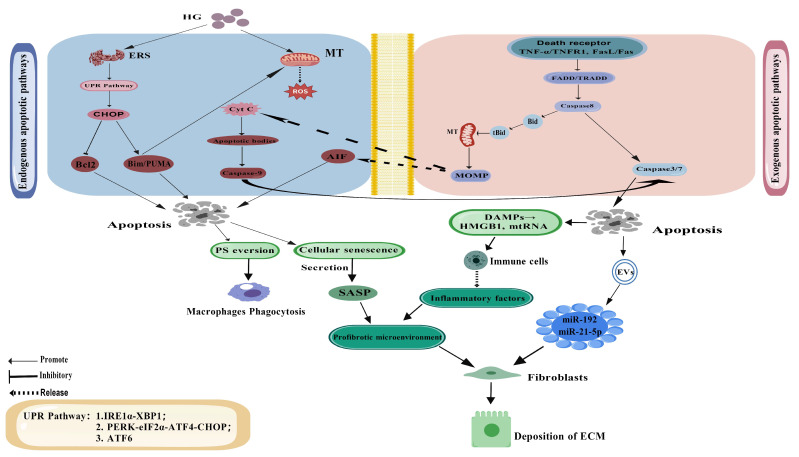
Signaling pathways and microenvironmental impact of PTECs apoptosis in DN. High glucose triggers PTECs apoptosis through extrinsic (death receptor-mediated, e.g., TNF-α/TNFR, FasL/Fas) and intrinsic (mitochondria/ER stress-mediated) pathways. Mitochondrial dysfunction leads to MOMP, releasing cytochrome c (cyt c) and activating caspases. ER stress via the UPR (PERK-eIF2α-ATF4-CHOP, IRE1α-ASK1-JNK) also promotes apoptosis. Apoptotic PTECs actively contribute to microenvironment remodeling by releasing DAMPs (e.g., HMGB1, mtDNA), which exacerbate inflammation via TLRs/NLRP3, and by exposing phosphatidylserine (PS) (“eat-me” signal) for phagocytic clearance. Additionally, they release extracellular vesicles (EVs) containing miRNAs (e.g., miR-21-5p, miR-192) that promote fibroblast activation and ECM synthesis in neighboring cells, thereby fueling a vicious cycle of apoptosis–inflammation–fibrosis. Solid arrows indicate activation or promotion; dashed arrows indicate translocation or release. TNFR, TNF receptor; FasL, Fas ligand; MOMP, mitochondrial outer membrane permeability; cyt c, cytochrome c; UPR, unfolded protein response; CHOP, C/EBP homologous protein; DAMPs, damage-associated molecular patterns; HMGB1, high mobility group box 1; mtDNA, mitochondrial DNA; PS, phosphatidylserine; EVs, extracellular vesicles; ECM, extracellular matrix.

The extrinsic apoptotic pathway is mainly mediated by DR and their ligands on the cell membrane and is particularly active in the inflammatory microenvironment of DN. TNF-α, Fas ligand (FasL), and TNF-related apoptosis-inducing ligand (TRAIL) bind to their corresponding receptors (TNFR1, Fas, and DR4/5) [70,71,72] to trigger the aggregation of intracellular death domains, recruit adaptor proteins such as Fas-associated protein with death domain (FADD) and TNF Receptor 1-associated death domain protein (TRADD), and then activate caspase-8. Activated caspase-8 can directly cleave the downstream effector caspase-3/7 and induce cell apoptosis [73]. It can also cleave BH3-interacting domain death agonist (Bid) protein to generate tBid, which translocalizes to mitochondria and induces increased mitochondrial outer membrane permeability (MOMP), thereby amplifying the apoptotic signal [74]. Recent studies have found that the expression of DR on the surface of PTECs is significantly upregulated in high-glucose environments. For example, the more classic Kelly et al. study in the (mRen-2)27 transgenic rat model revealed that hyperglycemia upregulates Fas protein expression in renal tubular cells, thereby promoting FasL-mediated apoptosis and accelerating the progression of diabetic nephropathy [75]. Furthermore, Su et al. showed that hyperglycemia upregulates key apoptotic receptors such as FAS and TNFR1 and activates downstream caspase pathways, leading to the death of renal tubular epithelial cells. The study also found that liraglutide, a Glucagon-like peptide-1 (GLP-1) receptor agonist, was able to mitigate this lipotoxic environment by inhibiting lipid synthesis and promoting lipolysis, thereby reducing lipid-induced renal tubular cell apoptosis. This suggests that glucolipid toxicity mediated by receptors such as FAS/TNFR1 plays a key role in renal tubular apoptosis, and targeting lipid metabolism may become an effective therapeutic strategy [76]. The initiation of this extrinsic pathway by death ligands is a key component of the apoptotic signaling network shown in (Figure 2).

More notably, the pattern of apoptosis may be dynamically switched in the high-glucose microenvironment. When caspase-8 activity is inhibited, PTECs may turn to necroptosis, a form of programmed necrosis activated by RIPK1 and RIPK3 kinases that ultimately leads to Mixed Lineage Kinase Domain-Like Protein (MLKL) oligomerization and cell membrane disruption [77]. Necroptosis releases a large number of DAMPs, such as HMGB1, Adenosine Triphosphate (ATP), and S100 Calcium-Binding Protein Family (S100), which strongly activates the immune response and promotes the spread of inflammation [8,78]. For instance, a study by Yu et al. revealed that co-exposure of high glucose and free fatty acids in diabetic nephropathy synergistically induces tubular epithelial cell injury, a process that involves the activation of RIPK3 and p-MLKL, key executive molecules of necroptosis. This necroptosis significantly exacerbates tubular injury and inflammatory cell infiltration, thereby contributing to the progression of nephropathy [79]. Kang et al. revealed that RIPK3 is activated and phosphorylates MLKL under high glucose, which disrupts mitochondrial function in podocytes through the PGAM5-Drp1 signaling pathway. This disruption leads to excessive mitochondrial fission, impaired energy metabolism, and ultimately podocyte injury and proteinuria. Although the primary focus has been on podocyte injury in diabetic nephropathy, its findings also provide important insights into the mechanisms of renal tubular injury in a high-glucose environment [80]. This suggests the potential of necroptosis-related proteins as biomarkers and therapeutic targets for disease progression.

In addition to traditional death receptors, some emerging regulatory molecules have also received attention. For example, TNF receptor-associated factor 2 (TRAF2) affects the balance between cell survival and death by regulating cIAP1/2 stability [81]. Bradford et al. have shown that apoptosis of PTECs during kidney injury is closely related to the depletion of TNIK (TRAF2 and NCK-interacting kinase). Deletion of TNIK leads to a decrease in the stability of TRAF2 protein, which in turn significantly promotes the apoptotic signaling pathway by enhancing the activation of caspase-8. At the same time, the abnormal activation of the NF-κB pathway and the release of pro-inflammatory factors further aggravate the microenvironment of inflammatory death of tubular cells [82]. This mechanism highlights the critical role of TRAF2 in regulating the balance between survival and apoptosis of renal tubular cells and provides a new perspective for understanding the molecular basis of tubular injury in diabetic nephropathy.

### 3.2. Endogenous Apoptotic Pathway: The Central Role of Mitochondria and Endoplasmic Reticulum Stress

The intrinsic apoptotic pathway is initiated from within the cell, primarily by MT dysfunction and severe ERS. This pathway integrates various internal damage signals, such as oxidative stress and metabolic disturbances.

The endogenous apoptosis pathway reflects the metabolic disorder and organelle dysfunction of PTECs under a high-glucose environment, in which MT and ER play a central role. Mitochondria, as the main site of cellular energy metabolism and ROS generation, are functionally severely impaired in DN [83]. High glucose induces mtDNA damage and membrane potential collapse by inhibiting the activity of electron transport chain complexes I and III, leading to superoxide overproduction [84]. The accumulation of ROS further activates pro-apoptotic proteins of the Bcl-2 family (such as Bax and Bak), which promote increased mitochondrial outer membrane permeability and release of cytochrome c (cyt c), Second mitochondria-derived activator of caspase/Direct IAP Binding Protein with Low pI (Smac/DIABLO), and apoptosis inducing factor (AIF) to the cytosol [85,86,87,88,89]. cyt c binds to Apoptotic protease-activating factor 1 (Apaf-1) to form apoptotic bodies, which activate caspase-9 and then initiate the cascade of caspase-3/7 [90]. AIF directly translocate to the nucleus and cause DNA fragmentation [91]. This process is independent of caspase and may be involved in some atypical apoptotic pathways. The central role of mitochondrial permeabilization in executing intrinsic apoptosis is depicted in (Figure 2).

Recent studies have highlighted the important role of mitochondrial quality control imbalance in PTECs apoptosis. For example, defects in mitophagy lead to accumulation of damaged mitochondria, exacerbating ROS production and amplification of apoptotic signals. A recent study Zheng et al. showed that the Jinchan Yishen-Tongluo recipe enhanced mitophagy and reduced cell damage by activating the HIF-1α-PTEN-Induced Kinase 1 (PINK1)-Parkin signaling pathway to alleviate MT dysfunction and PTECs apoptosis in diabetic nephropathy. Mechanically, this study further confirmed that specific deletion of PINK1, a key mitophagy protein, in PTECs significantly aggravated tubular apoptosis and fibrosis under diabetic conditions, highlighting the key role of PINK1-mediated mitophagy in protecting PTECs from high-glucose injury [92]. In contrast, the use of mitochondrial antioxidants (such as Mito-TEMPO [93]) or drugs that promote mitophagy (such as urolycin A [94]) significantly attenuated high glucose-induced apoptosis.

ERS is another important driver of endogenous apoptosis. In the DN state, unfolded or misfolded proteins accumulate in the endoplasmic reticulum lumen, triggering the UPR. Three branching pathways of the UPR (IRE1α-XBP1, PERK-eIF2α, and ATF6) are protective under short-term stress, but sustained activation turns to pro-apoptosis. The PERK-eIF2α-ATF4 pathway ultimately upregulates the pro-apoptotic factor CHOP, which exacerbates mitochondrial apoptosis by inhibiting the expression of the anti-apoptotic protein Bcl-2 and promoting the transcription of Bim and Proline-rich Ubiquitous Mitochondrial Antioxidant (PUMA) [95]. The IRE1α pathway can promote apoptosis by activating the ASK1-JNK pathway [96]. In a study by Liu et al., it was found that high glucose can lead to an abnormal increase in the ribonuclease activity of IRE1α in PTECs, which enhances the splicing of XBP1, which in turn activates the NLRP3 inflammasome and exacerbates renal inflammation and tubular damage. We further revealed that PDIA4, as an endogenous inhibitor, directly interacts with IRE1α to inhibit its rnase activity and decrease sXBP1 level, which ultimately attenuates the activation of the NLRP3 inflammasome, apoptosis, and fibrosis [61]. This study provides a new perspective to understand the protective mechanism of the PDIA4-IRE1α-sXBP1 axis in diabetic nephropathy, suggesting that this pathway may be a potential therapeutic target. The contribution of ER stress to apoptosis is also integrated into the signaling network shown in (Figure 2).

In addition, the role of Mitochondria-Associated (Endoplasmic Reticulum) Membranes (MAMs) in the regulation of apoptosis has been gradually revealed. MAMs are critical regions for calcium exchange and lipid metabolism, and their stability affects cell survival [97]. A study by Li et al. pointed out that the MAMs of PTECs in a diabetes model were structurally disordered and had an imbalance in calcium homeostasis, leading to mitochondrial calcium overload and enhanced apoptosis [98]. Restoring MAM integrity, such as through Mfn2 overexpression, improves cell survival, providing a new target for DN therapy.

### 3.3. Apoptotic PTECs as Active Participants in Microenvironment Remodeling

Apoptosis is not merely a “silent” process of cell removal. Dying PTECs actively release signaling molecules and undergo surface changes that profoundly influence neighboring cells and the tissue microenvironment, thereby driving disease progression.

The traditional view is that apoptosis is a “quiet” way of cell death. However, recent studies have found that apoptotic PTECs can actively regulate the renal microenvironment through a variety of mechanisms to promote the progression of DN. Firstly, DAMPs (such as HMGB1, mtDNA, heat shock proteins) released by apoptotic cells induce inflammatory responses in macrophages and fibroblasts by activating TLRs and the NLRP3 inflammasome [99,100]. For example, HMGB1 activates the NF-κB pathway after binding to TLR4 and promotes the secretion of TNF-α, IL-1β, and other cytokines, forming a positive feedback loop of inflammation [101]. A molecular pharmacology study by Yuan et al. showed that dapagliflozin attenuates diabetic kidney injury by inhibiting a self-perpetuating inflammatory cycle mediated by HMGB1 feedback signaling in the kidney. Dapagliflozin can reduce the expression and release of HMGB1 induced by high glucose in renal tubular epithelial cells, thereby inhibiting its interaction with TLR4 and subsequent activation of the NF-κB pathway, ultimately reducing the production of pro-inflammatory cytokines and improving renal fibrosis [102]. This DAMP-mediated communication from apoptotic cells to immune cells is a key feature of the microenvironmental impact illustrated in (Figure 2).

Second, membrane phosphatidylserine (PS) eversion during apoptosis mediates phagocytic clearance of macrophages via “eat-me” signals such as Annexin A5 and MFGE8 [103]. However, in the chronic inflammatory background of DN, macrophages tend to polarize into the profibrotic M2 type, secreting TGF-β1 and PDGF, stimulating myofibroblast activation and ECM deposition [104]. A histopathological study by Araujo et al. showed that the apoptotic index was significantly increased in the renal tissue of Leishmania-infected rats, accompanied by the upregulation of chemokines such as MCP-1 and C-X-C Motif Chemokine Ligand 10 (CXCL10) and the extensive infiltration of monocytes/lymphocytes, which were closely related to the damage of renal function and the severity of clinical symptoms [105].

In addition, extracellular vesicles (EVs) released by apoptotic PTECs play an important role in intercellular communication. These EVs carry proteins, lipids, and nucleic acids such as mirnas that can be taken up by neighboring cells, altering their phenotype and function [106]. For example, Mir-21-5p-enriched EVs activate the AKT/mTOR pathway by inhibiting PTEN expression and promote fibroblast proliferation and ECM synthesis. However, EVs carrying miR-214 aggravate mitochondrial dysfunction by inhibiting atpase activity [107]. A study by Jia et al. found that the exosomal release of miR-192 by PTECs under high glucose was significantly increased, and this miRNA promoted renal interstitial fibrosis by targeting Zinc Finger E-Box Binding Homeobox 2 (ZEB2) and enhancing the TGF-β1/Smad signaling pathway. Exendin-4 treatment could inhibit the secretion of miR-192, thereby reducing the progression of fibrosis [108]. This EV-mediated crosstalk is another critical mechanism by which apoptotic PTECs influence their surroundings, as summarized in (Figure 2).

Recent studies have also focused on the association between apoptotic cells and cellular senescence. Senescent PTECs recruit immune cells and promote fibrosis by releasing IL-6, MCP-1, and Matrix Metalloproteinases (MMPs) through the senescing-associated secretory phenotype (SASP) [109]. A study by Liu et al. showed that loss of p16INK4a significantly reduced renal fibrosis in obesity-related nephropathy by regulating metabolic reprogramming of renal cells and inhibiting the NLRP3 inflammasome pathway [110]. Andrographolide, a natural diterpenoid, attenuates renal fibrosis by mitigating lipotoxicity-induced premature senescence in renal tubular epithelial cells, thereby reducing senescence-associated secretory phenotype (SASP) and extracellular matrix deposition [111]. The signaling pathways of apoptosis and its profound impact on the renal microenvironment are comprehensively illustrated in (Figure 2).

## 4. Dynamic Evolution of PTECs Fibrosis and Remodeling of Microenvironment

Renal tubulointerstitial fibrosis (TIF) is the key pathological basis for the continuous deterioration of renal function and the eventual progression to end-stage renal disease in DN. Fibrosis is traditionally considered to be a result of passive repair after injury. However, recent studies have revealed that PTECs show a highly dynamic and active regulatory role in the process of fibrosis [112]. PTECs not only serve as direct responses to high glucose and inflammatory signals but also play an important role in the formation and evolution of the fibrotic microenvironment through phenotype switching, intercellular communication, and mechanical signal perception. The application of single-cell transcriptomics and spatial multi-omics technology further revealed that PTECs have obvious functional heterogeneity and spatiotemporal dynamic changes during fibrosis, and some subsets (such as CD44hi/ITGB1hi cells) even have “pro-fibrotic memory”. It can lead to abnormal deposition of ECM and tissue architecture destruction under the continuous stimulation of disease [113,114]. Fibrosis is not a linear irreversible process but is reversible within a certain time window. Its mechanism involves the plasticity of EMT, metabolic reprogramming, and dynamic changes in the mechanical microenvironment [115]. Therefore, a deep understanding of the multifunctional role of PTECs and their microenvironment interaction network in fibrosis is of great significance for the development of therapeutic strategies for reversing fibrosis.

### 4.1. Double-Edged Sword Effect of TGF-β1 Signaling Pathway

TGF-β1 is a pivotal cytokine that regulates cell growth, differentiation, and immune responses. In the context of kidney disease, it is the most potent pro-fibrotic factor, driving the excessive deposition of ECM.

TGF-β1 is widely considered to be the core regulator of renal fibrosis. The signal transduction of Tgf-β1 in PTECs is not limited to the classical Smad pathway but also involves multiple non-Smad signal crosstalk, which determine the process and reversibility of fibrosis [116]. Under the multiple stimulations of high glucose, inflammatory factors, and mechanical stress, PTECs produced a large amount of TGF-β1 through autocrine and paracrine mechanisms and then maintained their signaling activity in an autoinducible manner [117,118,119]. The classical Smad2/3 phosphorylation pathway promotes the degradation of transcriptional co-repressors such as SnoN and Ski, releasing the Smad complex into the nucleus and upregulating the expression of fibrosis-related genes such as α-smooth muscle actin (α-SMA), Fibronectin, and collagen I/III. At the same time, it inhibited the expression of epithelial marker E-cadherin and promoted the EMT process [120,121,122].

**Figure 3:**
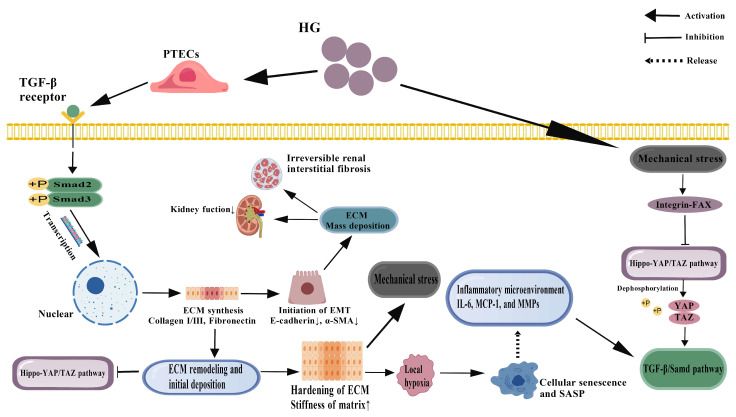
Interplay of inflammation, apoptosis, and fibrosis in PTECs under hyperglycemic conditions in diabetic nephropathy. High glucose induces metabolic disturbances in proximal tubular epithelial cells (PTECs), leading to oxidative stress, advanced glycation end products (AGEs) accumulation, and endoplasmic reticulum stress (ERS). These events activate key inflammatory pathways such as NF-κB and the NLRP3 inflammasome, resulting in the release of pro-inflammatory cytokines (e.g., TNF-α, IL-1β, IL-6) and damage-associated molecular patterns (DAMPs). The inflammatory microenvironment promotes apoptosis via death receptor and mitochondrial pathways. Apoptotic PTECs further release DAMPs, which exacerbate inflammation and activate fibroblasts, thereby promoting extracellular matrix (ECM) deposition and fibrosis. The fibrotic microenvironment, in turn, enhances mechanical stress and hypoxia, which feedback to amplify inflammation and apoptosis, forming a self-sustaining vicious cycle that drives the progression of diabetic nephropathy. Solid arrows indicate activation or promotion; dashed arrows indicate translocation or release.

However, recent studies have found that the output of TGF-β1 signaling has a clear background dependence and dose effect. Low concentrations of TGF-β1 tended to activate the Smad pathway and mediate reversible myofibroblast transdifferentiation and slight ECM accumulation. Nevertheless, high concentration or continuous stimulation strongly activates non-Smad pathways [123], including ERK, JNK, p38 MAPK, and PI3K/Akt/mTOR, which further amplify the fibrotic response and promote ECM cross-linking and stabilization by phosphorylating transcription factors such as AP-1, NF-κB, and STAT3 [124,125,126,127]. For instance, sustained activation of the ERK pathway enhances the stability of EMT transcription factors such as Zinc Finger Protein SNAI1 (Snail) and ZEB1, thereby reinforcing the loss of epithelial identity [128]; JNK promotes TGF-β1 expression through c-Jun phosphorylation, forming a positive feedback loop [129]. It is worth noting that TGF-β1 can also affect fibrosis progression by regulating the expression of micrornas, such as miR-21 and miR-192. miR-21 further enhanced Smad2/3 phosphorylation by inhibiting Smad7 [130], an endogenous inhibitor of TGF-β1 signaling, while miR-192 promoted EMT by downregulating ZEB2 [131]. The central position of TGF-β1 in coordinating the fibrotic response is highlighted in (Figure 3).

In addition, the synergistic effect of TGF-β1 with other cytokines such as Connective Tissue Growth Factor (CTGF) and Platelet-Derived Growth Factor (PDGF) cannot be ignored [132]. Together, these factors constitute a pro-fibrotic network that significantly enhances ECM production and inhibits its degradation. However, TGF-β1 signaling also has a negative feedback regulation mechanism, such as Smad7 and BAMBI (BMP and Activin Membrane-Bound Inhibitor), which can inhibit its excessive activation, suggesting that the TGF-β1 signaling pathway has a certain self-limiting ability [133]. Therefore, the intervention of TGF-β1 signaling needs to balance its dual role, and the ideal strategy should be fine regulation rather than complete inhibition, so as to balance the anti-fibrosis effect and tissue repair.

### 4.2. Mechanobiological Regulation of Hippo-YAP/TAZ Pathway

The Hippo signaling pathway is a conserved kinase cascade that controls organ size and cell proliferation. Its downstream effectors, YAP and TAZ, are key sensors and mediators of mechanical signals from the cellular microenvironment, such as ECM stiffness.

In recent years, the role of the Hippo-YAP/TAZ signaling pathway in renal fibrosis has received increasing attention as a key mediator for cells to sense and respond to the mechanical microenvironment. Under normal physiological conditions, Hippo pathway kinases (Mammalian Ste20-like kinase 1/2 (MST1/2) and Large Tumor Suppressor kinase 1/2 (LATS1/2)) phosphorylate and inhibit the transcriptional co-activators YAP and TAZ, promoting their cytosolic retention and degradation [134]. However, under conditions of high glucose, ECM stiffness, and altered cell–matrix interactions, this pathway is often inhibited, leading to YAP/TAZ dephosphorylation and translocation into the nucleus, where it binds to transcription factors such as Transcriptional Enhancer Activator Domain family (TEAD) to activate profibrotic gene programs [135,136,137]. As mechanosensitive cells, PTECs sense the physical properties of the surrounding matrix (such as stiffness and topology) through integrin-focal adhesion complexes, thereby regulating YAP/TAZ activity [138]. Progressive ECM hardening in fibrotic kidneys has been shown to enhance Rho-GTPase activity through activation of integrin-linked kinase (ILK) and focal adhesion kinase (FAK), which in turn inhibit LATS1/2 and promote YAP/TAZ nuclear translocation [139]. Once in the nucleus, YAP/TAZ directly upregulates multiple fibrosis-related factors, such as CTGF, cellular osteopontin (OPN), and plasminogen activator inhibitor-1 (PAI-1), while synergia with TGF-β1 signal to enhance EMT and ECM synthesis [140]. This mechano-sensitive pathway is a critical component of the fibrogenic network illustrated in (Figure 3).

Notably, YAP/TAZ activation not only responds to the mechanical properties of the static matrix but also dynamically regulates cytoskeletal reorganization and contractility, forming a cell-autonomous mechanical positive feedback loop. For example, YAP/TAZ activation can upregulate integrin expression and collagen cross-linking enzyme lysyl oxidase (LOX), which further increases matrix stiffness and thus activates YAP/TAZ more strongly [141]. This “stiffness-YAP-ECM stiffening” cycle is considered to be one of the central mechanisms of fibrosis self-maintenance. In addition, loss of cell polarity proteins (such as Crumbs Cell Polarity Complex Component 3 (CRB3) and Angiomotin (AMOT)) or disruption of tight junctions (such as Zonula Occludens-1 (ZO-1) degradation) can also unanchor the cytosol to YAP/TAZ and promote its nuclear translocation [142,143].

Recently, Wang et al. demonstrated that 14-3-3ζ protein could attenuate maladaptive tubular repair and fibrosis by directly binding to and inhibiting the transcriptional coactivator YAP, blocking its nuclear translocation and profibrotic activity. This interaction is disrupted in the pathological high-stiffness microenvironment, leading to the abnormal activation of YAP signaling and promoting renal tubular injury and extracellular matrix overdeposition. Notably, enhancing 14-3-3ζ expression by genetic or pharmacological means in experimental models can effectively inhibit YAP activation and improve renal interstitial fibrosis, suggesting that targeting the 14-3-3ζ/YAP signaling axis may be a new approach for anti-fibrosis treatment [144]. In addition, YAP/TAZ are also involved in the regulation of cell metabolism and autophagy, affecting the survival and phenotypic transformation of PTECs under stress conditions, further expanding the regulatory dimension of Ptecs in fibrosis [25].

### 4.3. Temporal and Spatial Heterogeneity of Fibrosis Progression and Window of Reversibility

Renal fibrosis is not a uniform process across the entire kidney. It exhibits significant spatial (location-specific) and temporal (time-dependent) heterogeneity, meaning it starts and progresses at different rates in different nephron segments. Importantly, early-stage fibrosis may be reversible, while late-stage, mature scar tissue is often permanent.

Renal tubulointerstitial fibrosis is a dynamic process with significant spatial and temporal heterogeneity. Its development is not uniform and synchronous but shows different initiation time, progression rate, and reversal potential in different nephron regions or even single tubular segments. This heterogeneity is due to the cellular heterogeneity of PTECs, local microenvironmental differences (such as oxygen partial pressure, inflammatory cell infiltration, capillary density), and epigenetic regulation [145]. In recent years, single-cell transcriptome and molecular mechanism studies have gradually revealed that PTECs have obvious heterogeneity and pro-fibrotic subsets in chronic kidney disease (CKD), including DN. For example, lncRNA MALAT1 promotes renal interstitial fibrosis by regulating the miR-124-3p/ITGB1 axis, suggesting that a subset of PTECs with high ITGB1 expression may have a stronger propensity to fibrosis [146]. In addition, Fu et al. identified multiple chronic kidney disease-associated fibrosis genes that were significantly enriched in the TGF-β signaling pathway and the extracellular matrix receptor interaction pathway, further supporting the involvement of a specific subset of PTECs in fibrosis maintenance through high expression of integrins such as ITGB1 and collagen [147]. Meanwhile, the complex evolution of MIF signaling also suggests that there is multi-level crosstalk between inflammation and fibrosis [148], which may affect the senescence and phenotypic transformation of PTECs, such as the formation of aging-like subsets with high expression of p16INK4a. Together, these subpopulations constitute “holdout sites” for fibrosis progression, whereas others may retain some regenerative capacity.

The early stage of fibrosis is characterized by reversible changes, which are characterized by the accumulation of a small amount of activated myofibroblasts, deposition of type III collagen, and local inflammation in the renal interstium [149]. At this point, if the pathogenic factors (such as high glucose) are removed in time or targeted interventions (such as SGLT2 inhibitors and YAP/TAZ inhibitors) are applied, the degree of fibrosis can be significantly reduced or even reversed [150]. The mechanisms may involve myofibroblast apoptosis, enhanced ECM degradation (via activation of MMPs), and restoration of the epithelial properties of PTECs. However, as the disease progresses, the ECM undergoes dramatic remodeling: collagen fibers become cross-linked (catalyzed by LOX family enzymes), the composition of proteoglycan and glycoaminoglycan is altered, and elastic fibers are lost, resulting in a highly stable and resistant matrix network. This structure not only hinders cell migration and tissue repair but also continuously activates pro-fibrotic pathways through integrin signaling and causes microvascular sparse and tissue hypoxia, which induces abnormal expression of VEGF and pericyte loss through HIF-1α, further exacerbating the vicious cycle between fibrosis and ischemia [151]. The transition from a reversible inflammatory state to irreversible fibrosis is a key concept depicted in the cycle shown in (Figure 3).

In addition, senescence plays a key role in the irreversible transition of fibrosis: senescent PTECs release a large number of inflammatory factors, chemokines, and matrix-degrading enzyme inhibitors through the senesence-associated secretory phenotype (SASP), actively create a pro-fibrotic microenvironment, and resist apoptosis, thereby continuously driving disease progression for a long time [152]. Time-resolved lineage tracing and in vivo imaging studies have further revealed that there is a time window for regression of fibrosis beyond which fibrosis may progress autonomously even after the initial injury-causing factors have been eliminated [153,154]. Therefore, identifying and targeting this window of reversibility, as well as eliminating a subset of persistently activated profibrotic cells, will be important directions for future therapeutic strategies. The intricate crosstalk between inflammation, apoptosis, and fibrosis, which forms a self-sustaining vicious cycle driving DN progression, is depicted in (Figure 3).

## 5. Interaction of Inflammation–Apoptosis–Fibrosis Network

**Figure 4:**
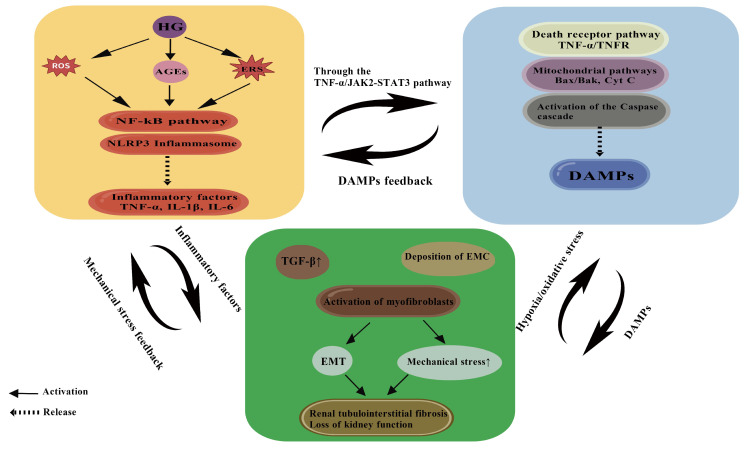
The self-reinforcing vicious cycle of the inflammation–apoptosis–fibrosis crosstalk in PTECs drives the progression of diabetic nephropathy. This schematic illustrates the intricate and cyclical interplay between three core pathological processes in proximal tubular epithelial cells (PTECs) under hyperglycemic conditions. (1) Initiation: Hyperglycemia-induced metabolic disturbances (e.g., oxidative stress, AGE accumulation, ER stress) primarily trigger an inflammatory response via activation of key pathways like NF-κB and the NLRP3 inflammasome, leading to the release of pro-inflammatory cytokines (TNF-α, IL-1β, IL-6). (2) Amplification: The inflammatory milieu promotes PTECs apoptosis through both extrinsic (e.g., death receptor) and intrinsic (e.g., mitochondrial) pathways. Apoptotic cells release damage-associated molecular patterns (DAMPs, e.g., HMGB1), which further potentiate inflammation and immune cell activation, creating a positive feedback loop between inflammation and apoptosis. (3) Fibrotic Transformation: Inflammatory cytokines and DAMPs activate fibroblasts and upregulate profibrotic factors like TGF-β1. TGF-β1, in concert with other signaling pathways (e.g., JAK2/STAT3, RhoA/ROCK), drives epithelial–mesenchymal transition (EMT) and excessive extracellular matrix (ECM) deposition, culminating in renal fibrosis. (4) Cycle Perpetuation: The established fibrotic microenvironment, characterized by ECM stiffness, hypoxia, and altered mechanical stress, feedbacks to exacerbate oxidative stress, inflammation, and apoptosis, thereby forming a self-sustaining vicious cycle that propels diabetic kidney disease toward end-stage renal failure. Solid arrows indicate direct activation or promotion; dashed arrows represent translocation, release, or secondary effects.

As summarized (Figure 4), a self-reinforcing vicious cycle is formed among inflammation, apoptosis, and fibrosis, collectively driving the progression of DN. In the pathological process of DN, inflammation, apoptosis, and fibrosis do not exist in isolation but form a complex interaction network to promote the progress of the disease. When the dynamic balance of this network is broken, various pathological processes promote each other and form a vicious circle, eventually leading to renal tubulointerstitial fibrosis and irreversible damage to renal function. As the core participants in this network, the injury mechanism of PTECs is closely related to the interaction among the three. As the initial factor of DN, hyperglycemia induces the excessive production of ROS by activating the polyol pathway, PKC pathway, and the generation of AGEs and then triggers an inflammatory response [155,156]. The activation of inflammatory response is mainly mediated by the NF-κB signaling pathway, which promotes the release of inflammatory factors such as TNF-α and IL-1β. These factors not only directly damage PTECs but also promote apoptosis by activating apoptotic-related pathways such as TNF-α/TNFR and JAK2/STAT3. Apoptotic PTECs further release DAMPs, such as HMGB and heat shock proteins, which activate immune cells through Pattern Recognition Receptors (PRRs) and exacerbate inflammatory responses, forming a vicious cycle of inflammation–apoptosis.

At the same time, inflammation and apoptosis jointly promote the process of fibrosis. Inflammatory factors such as TNF-α and IL-1β can upregulate the expression of TGF-β1, which is a core regulator of fibrosis. The expression of PI3K and renin-angiotensin system (RAS) homolog family member A/Rho-associated coiled-coil proteins was detected by Western blot coiled-coil-containing protein kinase (RhoA/ROCK) pathway, which activates myofibroblast differentiation and promotes the deposition of ECM [157]. In addition, DAMPs released by apoptotic cells can also directly activate fibroblasts and accelerate fibrosis formation. Fibrosis is not merely a passive consequence of inflammation and apoptosis but it can also enhance inflammation and apoptosis by feedback through a variety of mechanisms. For example, excessive deposition of ECM in fibrotic tissue can lead to increased tissue mechanical stress, which further activates TGF-β1 and NF-κB signaling pathways and promotes the release of inflammatory factors. At the same time, the aggravation of hypoxia and oxidative stress in the fibrotic microenvironment induces apoptosis of PTECs through mitochondrial dysfunction and endoplasmic reticulum stress. This interaction forms a positive feedback loop in which inflammation, apoptosis, and fibrosis are intertwined to jointly drive the progression of DN to ESRD.

In this network, metabolic disorders and signaling pathway abnormalities of PTECs play a key role. Impaired mitochondrial function of PTECs in a hyperglycemia environment leads to a large accumulation of ROS, which not only directly activates inflammatory and apoptotic pathways but also further aggravates cell damage by promoting the formation of AGEs. After binding to its receptor RAGE, AGEs activate the NF-κB and MAPK pathways, amplify inflammatory response, and promote fibrosis by upregulating the expression of TGF-β1. In addition, hyperglycemia also enhances the transcription and secretion of inflammatory factors by activating the hexosamine pathway and PKC isoforms, which further promotes the operation of the inflammation–apoptosis–fibrosis network. It is worth noting that the interaction of this network is not unidirectional or linear but shows a multi-level, multi-pathway complexity. For example, TGF-β1 can not only directly induce fibrosis but also indirectly aggravate inflammation and apoptosis by inhibiting autophagy and promoting cell senescence. The inflammatory factor IL-6 is involved in the regulation of apoptosis and fibrosis by activating the JAK2/STAT3 pathway [158].

## 6. Treatment Strategy: From Molecular Mechanism to Clinical Translation

The understanding of the intertwined inflammation–apoptosis–fibrosis network in PTECs has driven a paradigm shift in DN therapeutic strategy, from purely glycemic control to targeted multi-pathway interventions. This network’s complexity necessitates diverse pharmacological approaches. Broadly, these can be categorized into two complementary strategies: (1) monotargeted agents, typically Western-developed drugs designed for high specificity against a single key node in the pathological network, such as mineralocorticoid receptor (MR), SGLT2, NLRP3 inflammasome and fibrosis-related factors [159,160,161,162]; and (2) multi-targeted formulations, often derived from natural products or traditional medicines like TCM, which aim to synergistically modulate multiple pathways simultaneously. The following sections evaluate the evidence for these strategies, not based on their origin but on their pharmacological rationale and their ability to disrupt the vicious cycle in PTECs.

### 6.1. Monotargeted Agents: Precision and Specificity

In the field of anti-inflammatory treatment, research on Western drugs has progressed beyond traditional RAS inhibitors toward more specific molecular targets. For instance, non-steroidal mineralocorticoid receptor antagonists (MRAs), such as finerenone, selectively block the MR. Moreover, finerenone has been shown to ameliorate mitochondrial dysfunction in diabetic renal tubules by activating the PI3K/Akt/eNOS signaling pathway, thereby attenuating oxidative stress and tubular injury [163]. Notably, the FIDELIO-DKD and FIGARO-DKD clinical trials demonstrated that finerenone reduced the primary composite renal outcome (kidney failure, sustained ≥40% eGFR decline, renal death) by 18% (hazard ratio [HR] 0.82; 95% CI, 0.73–0.93) over a median of 2.6 years compared to placebo and reduced urinary protein excretion rates and delaed renal function deterioration in DN patients, with anti-inflammatory effects independent of blood pressure control [164,165]. Furthermore, SGLT2 inhibitors—including empagliflozin and dapagliflozin—not only lower blood glucose but also inhibit inflammatory signaling pathways by improving energy metabolism in PTECs and reducing mitochondrial ROS production [165]. Importantly, large-scale trials such as EMPA-REG OUTCOME, DAPA-CKD, and CREDENCE have indicated that SGLT2 inhibitors significantly reduce the risk of cardiorenal events in DN patients, potentially through AMPK activation and downregulation of inflammatory factors. Among them, Empagliflozin treatment in the EMPA-REG OUTCOME trial led to a 39% relative risk reduction in incident or worsening nephropathy (HR 0.61; 95% CI, 0.53–0.70) [165,166,167,168]. Additionally, dapagliflozin has been found to delay renal tubular fibrosis in diabetic models by reducing high-glucose-induced ECM stiffness and abnormal mechanical stress accumulation, thereby inhibiting YAP/TAZ and TEAD-mediated profibrotic gene expression [169]. Meanwhile, genetic and pharmacological studies reveal that canagliflozin alleviates PTEC fibrosis and apoptosis by inhibiting Hhip-dependent Hedgehog signaling, thereby mitigating renal tubular damage in diabetic Akita mice [170]. Similarly, gemigliptin significantly attenuates renal fibrosis by suppressing NLRP3 inflammasome activation and subsequent IL-1β secretion in renal tubular cells. Of note, this protective effect is abolished upon NLRP3 overexpression, suggesting that targeting NLRP3-mediated inflammation may represent a novel therapeutic strategy for diabetic renal fibrosis [171].

In the context of kinase signaling, the JAK1/JAK2 inhibitor baricitinib significantly reduced the urinary albumin-to-creatinine ratio (UACR) in a phase 2 randomized controlled trial involving patients with type 2 diabetes and diabetic kidney disease. This finding provides clinical evidence supporting the targeting of the JAK/STAT pathway in DN treatment [172]. Likewise, the active vitamin D analog calcitriol attenuates PTECs apoptosis in DN by specifically inhibiting p38-MAPK phosphorylation, showing promise as an adjunct therapy. However, its potential effects on systemic calcium and phosphate metabolism warrant careful clinical consideration [173]. Additionally, simvastatin exhibits protective effects in a streptozotocin-induced type 1 diabetic rat model, primarily through potent antioxidant and anti-apoptosis mechanisms. These include upregulating renal antioxidant enzymes and modulating Bcl-2 family protein balance, thereby offering experimental support for its potential use in DN therapy [174]. Moreover, endoplasmic reticulum stress modulators such as 4-phenylbutyric acid (4-PBA) [175] and tauroursodeoxycholic acid (TUDCA) [176] mitigate high-glucose-induced PTECs apoptosis via suppression of the ATF4/CHOP pathway, presenting novel avenues for DN treatment. Finally, animal and clinical studies on GLP-1 receptor agonists (e.g., liraglutide) indicate that they confer renoprotection not only through metabolic improvements but also via direct anti-inflammatory and excretion-regulating effects. For example, liraglutide inhibits renal JAK/STAT signaling in db/db mice, thereby reducing PTEC inflammation and fibrosis. Clinical analyses further suggest that both short- and long-term liraglutide treatment enhances uric acid excretion and improves renal filtration, indicating tubular effects independent of glycemic control. Thus, resolving renal inflammation and hyperuricemia via GLP-1 agonists may offer new therapeutic strategies [177,178].

### 6.2. Multi-Targeted Formulations: Synergistic Network Modulation

The unique advantage of traditional Chinese medicine (TCM) in the treatment of DN lies in its synergistic effect of multiple components, which can interfere with inflammation, apoptosis, and fibrosis at the same time. Quercetin and Myricetin, the active ingredients of the Huangkui capsule, have been widely used in the clinical treatment of DN. The Huangkui capsule inhibits the apoptosis of PTECs by inhibiting PI3K/Akt and JAK2/STAT3 pathways and downregulates TGF-β1/Smad3 signaling to delay fibrosis [17,179,180]. As a primary active constituent of rhubarb, Emodin effectively attenuates the TGF-β1-induced EMT in renal tubular epithelial cells by suppressing the hyperactivation of the mTOR signaling pathway and the subsequent phosphorylation of its downstream effector, ribosomal protein S6 kinase (p70S6K) [181]. Astragaloside IV, a primary bioactive constituent of Astragalus membranaceus, has been demonstrated to alleviate high-glucose-induced apoptosis in renal tubular epithelial cells by upregulating the Bcl-2/Bax ratio and inhibiting Caspase-3 activation. Furthermore, it enhances the expression of mitophagy-related proteins PINK1 and Parkin, suggesting its role in promoting mitophagy and cellular protection [182]. Tanshinone IIA, a primary active compound derived from Salvia miltiorrhiza, has been demonstrated to effectively attenuate renal proximal tubular fibrosis by inhibiting the activation of the TGF-β1/Smad signaling pathway. This inhibition leads to a marked downregulation of fibrotic markers including α-SMA and type I collagen, while concurrently restoring the expression of the epithelial marker E-cadherin [183]. Tripterygium Glycosides Tablet ameliorates renal tubulointerstitial fibrosis in high-fat diet-fed and streptozotocin-induced diabetic rats by suppressing the TLR4/NF-κB signaling pathway, providing novel mechanistic insights into its potential for treating diabetic nephropathy. However, the risk of liver toxicity should be paid attention to [184]. Furthermore, Cordycepin, derived from Cordyceps sinensis, ameliorates renal interstitial fibrosis by inhibiting Drp1-mediated mitochondrial fission, thereby improving mitochondrial dysfunction and attenuating tubular epithelial cell apoptosis [185]. Puerarin, a natural compound derived from Kudzu root, ameliorates excessive extracellular matrix accumulation in diabetic nephropathy by inhibiting ferroptosis and downregulating the TGF-β1/Smad3 signaling pathway [186]. Baicalin ameliorates renal fibrosis through the microRNA-124/TLR4/NF-κB signaling axis in streptozotocin-induced diabetic nephropathy mice and high-glucose-treated human proximal tubule epithelial cells [187]. Ginsenoside Rg3 ameliorates high-fat diet/streptozotocin (HFD/STZ)-induced diabetic nephropathy by suppressing the MAPK/NF-κB signaling pathways, thereby attenuating renal inflammation and fibrosis [188]. Icariin prevents extracellular matrix accumulation and ameliorates experimental diabetic kidney disease by inhibiting oxidative stress via GPER-mediated p62-dependent Keap1 degradation and Nrf2 activation [189]. Spinach extract can reduce oxidative stress and inflammation and improve renal fibrosis and PTECs apoptosis by interfering with the AGEs/RAGE signaling axis, thereby alleviating renal injury in diabetic rats [190].

In addition to single TCM, compound preparations also show good prospects in the treatment of DN. In recent years, network pharmacology-based studies have further elucidated the multi-target mechanisms of traditional Chinese compound preparations, highlighting their promising prospects in the treatment of diabetic nephropathy and providing a scientific foundation for the modernization and global acceptance of TCM. For example, the Liuwei Dihuang Pill treats diabetic nephropathy in rats by inhibiting the TGF-β/SMADs, MAPK, and NF-κB signaling pathways and upregulating the expression of cytoglobin in renal tissues, thereby alleviating renal tubular and mesangial cell inflammation and fibrosis [191]. The Yi-Shen-Hua-Shi granule, on the other hand, ameliorates diabetic kidney disease via the “gut-kidney axis” mechanism by modulating the gut microbiota and enhancing intestinal barrier function, thereby mitigating systemic and renal inflammation. Its protective effects on glomerular podocytes and the renal tubulointerstitium significantly improve the pathological progression of DKD [192]. The Huangkui capsule (HKC) exerts tubulo-protective effects in DN through multi-target mechanisms. A multicenter clinical trial showed that the Huangkui capsule on top of RAS inhibition reduced proteinuria by approximately 30% more than placebo over 24 weeks [193]. Mechanistically, HKC induces mitophagy in renal tubular cells by activating the STING1/PINK1 signaling pathway, which promotes the clearance of damaged mitochondria and alleviates cellular oxidative stress and inflammation [194]. Shenyan Kangfu Tablets ameliorate renal tubular epithelial cell injury and tubulointerstitial fibrosis, thereby exerting a protective effect on the renal tubulointerstitium, by modulating the PI3K-Akt and HIF-1 signaling pathways and correcting the disturbances of key metabolites such as arginine and proline [195]. The Dang-Gui-Bu-Xue decoction exerts protective effects against diabetic nephropathy by modulating the carbonyl compounds’ metabolic profile and inhibiting the AGEs/RAGE pathway, thereby alleviating renal inflammation and fibrosis, ultimately preserving the structure and function of both the glomeruli and renal tubulointerstitium [196]. The Tangshen Formula alleviates inflammatory injury in aged diabetic kidney disease by modulating gut microbiota composition and related amino acid metabolism, thereby exerting a protective effect on the renal tubulointerstitium [197]. The Tangshenkang Granule exerts multi-faceted protective effects on renal tissues in diabetic nephropathy, specifically by mitigating mesangial matrix expansion in glomeruli and tubulointerstitial fibrosis. Its mechanisms are closely associated with improving lipid metabolism and suppressing oxidative stress and inflammatory responses [198]. As summarized (in Table 1), current pharmacological agents, both Western and Traditional Chinese Medicine, target various nodes within the inflammation–apoptosis–fibrosis axis, providing a multi-faceted approach to DN treatment.

### 6.3. Future Directions: Integrated Traditional Chinese and Western Medicine and Individualized Treatment

Although Western drugs have high specificity in targeted therapy, long-term use may face problems of drug resistance and side effects. Although traditional Chinese medicine has the advantage of multiple targets, its components are complex, and its standardization and mechanism research still need to be strengthened. Therefore, the future treatment strategy of DN may trend towards the integrated traditional Chinese and Western medicine model, such as combining Finerenone or Metformin with the Huangkui capsule, which can not only enhance the anti-inflammatory effect but also reduce the side effects of single-drug dose dependence [199]. In addition, individualized treatment based on biomarkers such as urinary kidney injury molecule-1 (KIM-1), neutrophil gelatinase-associated lipocalin (NGAL) or serum TGF-β1 levels will help optimize drug selection and improve efficacy [200,201]. With the development of gene editing and organoid technology, specific gene therapy for PTECs, such as CRISPR-Cas9-targeted editing of pro-fibrotic genes, may become possible [202]. Meanwhile, artificial intelligence-assisted drug screening can accelerate the discovery of novel anti-fibrotic compounds. For example, virtual screening based on deep learning has identified several natural compounds with potential renoprotective effects [203]. Other future directions include the development of drugs that target the metabolic reprogramming of PTECs, such as inhibitors of glycolysis (2-deoxyglucose) or promoters of fatty acid oxidation (such as L-carnitine) [204,205]; small molecules that target mitochondrial dynamics (such as the Drp1 inhibitor Mdavi-1) and mitochondrial–endoplasmic reticulum coupling (MAMs) [206]; and the use of nanotechnology to achieve kidney-targeted drug delivery, improve efficacy, and reduce systemic toxicity [207]. In conclusion, the treatment of DN has entered the era of multi-target and individualization, and the strategy of combining modern medicine with traditional Chinese medicine will provide a more comprehensive solution for delaying the progression of DN.

This table summarizes pharmacological agents discussed in Section 6 that target key pathways in the inflammation–apoptosis–fibrosis network of PTECs in diabetic nephropathy DN. Both Western medicines and TCM with demonstrated effects in preclinical or clinical studies are included.

**Table 1:** Monotargeted and multi-targeted pharmacological agents against the inflammation–apoptosis–fibrosis axis in diabetic nephropathy PTECs.

Types of Drugs	Name of Drug	Remarks/Mechanism Description	The Pathological Process of Action	Level of Evidence	Key Quantitative Outcomes
Western medicine	Finerenone [163,164,165]	Mineralocorticoid receptor (MR) → inhibits NF-κB/NLRP3 FIDELIO-DKD, FIGARO-DKD	Inflammation, Fibrosis	Preclinical/Clinical	18% reduction in primary composite renal outcome (HR 0.82; 95% CI, 0.73–0.93) in FIDELIO-DKD trial; Improved mitochondrial membrane potential and ATP production in HK-2 cells under high glucose
Western medicine	Empagliflozin [165,166]	SGLT2 → AMPK activation, mitochondrial ROS reduction EMPA-REG OUTCOME	Inflammation, Apoptosis, Fibrosis	Preclinical/Clinical	39% relative risk reduction in incident/worsening nephropathy (HR 0.61; 95% CI, 0.53–0.70) in EMPA-REG OUTCOME Reduced renal cortical ROS by ~40% in diabetic mice
Western medicine	Dapagliflozin [167,169]	SGLT2 → inhibits HMGB1/TLR4/NF-κB YAP/TAZ DAPA-CKD	Inflammation, Fibrosis	Preclinical/Clinical	36% reduction in UACR in DAPA-CKD trial Suppressed YAP/TAZ nuclear translocation by >50% in HG-treated HK-2 cells
Western medicine	Canagliflozin [168,170]	Inhibits Hhip-Hedgehog pathway CREDENCE	Apoptosis, Fibrosis	Preclinical/Clinical	30% reduction in kidney failure risk in CREDENCE trial Reduced tubular apoptosis by 45% in diabetic Akita mice
Western medicine	Gemigliptin [171]	NLRP3 inflammasome inhibitor	Inflammation	Preclinical/Early clinical	Reduced IL-1β secretion by ~60% in HG-stimulated HK-2 cells
Western medicine	Baricitinib [172]	JAK1/2 → inhibits JAK2/STAT3	Apoptosis, Inflammation	Preclinical	41% reduction in UACR in a Phase 2 RCT of T2D patients with DN
Western medicine	Simvastatin [173]	Nrf2 activation; inhibits NADPH oxidase and NF-κB	Apoptosis	Preclinical	Decreased renal caspase-3 activity by 35% in STZ-induced diabetic rats
Western medicine	Calcitriol [174]	Inhibits p38-MAPK phosphorylation	Apoptosis	Preclinical	Reduced tubular apoptosis by 40% in db/db mice via p38-MAPK inhibition
Western medicine	4-PBA [175]	ER stress inhibitor → ATF4/CHOP pathway	Apoptosis, ERS	Preclinical	Suppressed CHOP expression by 50% in HK-2 cells under ER stress
Western medicine	TUDCA [176]	ER stress modulator	Apoptosis, ERS	Preclinical	Reduced tubular apoptosis by 55% in diabetic mice models
Western medicine	Liraglutide [177,178]	Inhibits JAK2/STAT3 Enhance uric acid excretion → improve renal function	Inflammation, Fibrosis	Preclinical/Clinical	Increased uric acid excretion by 25% in clinical studies
Traditional Chinese Medicine	Quercetin [17,179]	Inhibits PI3K/Akt and JAK2/STAT3	Inflammation, Apoptosis, Fibrosis	Preclinical	Decreased caspase-3 activity by 50% in HG-induced HK-2 cells
Traditional Chinese Medicine	Myricetin [180]	Inhibits PI3K/Akt and TGF-β1/Smad3	Inflammation, Apoptosis, Fibrosis	Preclinical	Reduced TGF-β1-induced collagen I expression by 40% in NRK-52E cells
Traditional Chinese Medicine	Emodin [181]	Inhibits mTOR/p70S6K → suppresses EMT	Fibrosis	Preclinical	Inhibited mTOR/p70S6K phosphorylation by 60% in TGF-β1-treated HK-2 cells
Traditional Chinese Medicine	Astragaloside IV [182]	Upregulates Bcl-2/Bax, promotes PINK1/Parkin mitophagy	Apoptosis, Oxidative Stress	Preclinical/Clinical	Increased Bcl-2/Bax ratio by 2.5-fold in HG-treated HK-2 cells 188
Traditional Chinese Medicine	Tanshinone IIA [183]	Inhibits TGF-β1/Smad → suppresses EMT	EMT, Fibrosis	Preclinical	Reduced α-SMA expression by 50% in DN mice model
Traditional Chinese Medicine	Tripterygium glycosides [184]	Inhibits TLR4/NF-κB	Inflammation	Clinical (hepatotoxicity risk)	Suppressed TLR4/NF-κB pathway and reduced renal inflammation in diabetic rats
Traditional Chinese Medicine	Cordycepin [185]	Inhibits Drp1-mediated mitochondrial fission	Fibrosis, Apoptosis	Preclinical	Inhibited Drp1-mediated mitochondrial fission and reduced apoptosis by 40% in HK-2 cells
Traditional Chinese Medicine	Puerarin [186]	Inhibits ferroptosis and TGF-β1/Smad3	Fibrosis, Oxidative Stress	Preclinical	Suppressed TGF-β1/Smad3 activation and reduced fibronectin by 50% in db/db mice
Traditional Chinese Medicine	Baicalin [187]	miR-124/TLR4/NF-κB axis	Inflammation, Fibrosis	Preclinical	Downregulated TLR4/NF-κB and reduced renal IL-6 by 45% in STZ mice
Traditional Chinese Medicine	Ginsenoside Rg3 [188]	Inhibits MAPK/NF-κB	Inflammation, Fibrosis	Preclinical	Inhibited MAPK/NF-κB and reduced renal TNF-α by 50% in HFD/STZ mice
Traditional Chinese Medicine	Icariin [189]	Activates Nrf2 via GPER/p62/Keap1	Apoptosis	Preclinical	Activated Nrf2 and reduced ROS by 40% in HG-treated HK-2 cells
Traditional Chinese Medicine	Spinach extract [190]	Inhibits AGEs/RAGE axis	Inflammation, Apoptosis, Fibrosis	Preclinical	Reduced AGEs-induced RAGE expression by 35% in diabetic rat kidneys
Traditional Chinese Medicine	Huangkui capsule [191,192]	Multi-target: PI3K/Akt, JAK2/STAT3, TGF-β1/Smad3	Inflammation, Apoptosis, Fibrosis	Clinical/Preclinical	Reduced proteinuria by ~30% in a multicenter clinical trial Induced mitophagy via STING1/PINK1 in tubular cells
Traditional Chinese Medicine	Liuwei Dihuang pill [193]	Inhibits TGF-β/SMADs, MAPK, NF-κB; upregulates cytoglobin	Inflammation, Fibrosis	Preclinical	Downregulated TGF-β/SMADs and reduced renal fibrosis score by 40% in diabetic rats
Traditional Chinese Medicine	Yi-Shen-Hua-Shi granule [194]	Modulates gut microbiota → “gut-kidney axis”	Inflammation	Preclinical	Improved gut microbiota diversity and reduced renal IL-1β by 50% in DN rats
Traditional Chinese Medicine	Shenyan Kangfu Tablets [195]	Modulates PI3K-Akt and HIF-1 pathways	Tubular Injury, Fibrosis	Preclinical	Modulated PI3K-Akt/HIF-1 pathways and improved tubular injury score by 35% in DN rats
Traditional Chinese Medicine	Dang-Gui-Bu-Xue decoction [196]	Inhibits AGEs/RAGE pathway	Inflammation, Fibrosis	Preclinical	Inhibited AGEs/RAGE and reduced renal collagen IV by 40% in db/db mice
Traditional Chinese Medicine	Tangshen Formula [197]	Modulates gut microbiota and amino acid metabolism	Inflammation	Preclinical	Modulated gut microbiota and reduced renal MCP-1 by 45% in aged DN mice
Traditional Chinese Medicine	Tangshenkang Granule [198]	Improves lipid metabolism, suppresses oxidative stress and inflammation	Fibrosis	Preclinical	Improved lipid metabolism and reduced renal TGF-β1 by 40% in STZ rats

## 7. Conclusions and Prospects

In conclusion, this review synthesizes current evidence to establish a central thesis: the progression of DN is critically driven by a self-sustaining vicious cycle of inflammation, apoptosis, and fibrosis within PTECs. Hyperglycemia-induced metabolic insults initiate this cycle by activating key inflammatory pathways. The resulting inflammatory milieu promotes PTECs apoptosis, which in turn amplifies inflammation via DAMPs release. These processes collectively fuel a fibrotic response, and the stiffened, hypoxic fibrotic matrix then feedbacks to reinforce the initial inflammatory and apoptotic signals. This integrated pathomechanism underscores the limitation of mono-mechanistic therapies.

The primary conclusion from our analysis of the cited literature is that future therapeutic success in DN hinges on the development of strategies capable of disrupting this PTEC-centric pathological network. This can be achieved through two complementary avenues: (1) the rational combination of monotargeted agents (e.g., SGLT2 inhibitors, Finerenone) that potently inhibit key individual nodes, and (2) the development and standardization of multi-targeted formulations (e.g., refined TCM compounds) that inherently target multiple network components. The choice between or combination of these strategies should be guided by disease stage, patient phenotype, and robust biomarkers.

Future efforts must focus on the following: (1) leveraging single-cell multi-omics to deconvolute PTEC heterogeneity and identify novel druggable targets within specific subpopulations; (2) exploring metabolic and epigenetic modifiers to reset cellular phenotypes; (3) targeting the mechanobiological interface to reverse early fibrosis; and (4) advancing kidney-specific drug delivery systems to maximize efficacy and minimize systemic exposure. By embracing a network pharmacology approach and moving beyond traditional therapeutic silos, we can pave the way for more effective, personalized therapies to halt DN progression.

## Data Availability

The data presented in this study are openly available in BioGDP (https://doi.org/10.1093/nar/gkae973).
